# [2.2.2.2]Paracyclophanetetraenes (PCTs): cyclic structural analogues of poly( *p*‑phenylene vinylene)s (PPVs)

**DOI:** 10.12688/openreseurope.13723.1

**Published:** 2021-09-23

**Authors:** Matthias Pletzer, Felix Plasser, Martina Rimmele, Martin Heeney, Florian Glöcklhofer

**Affiliations:** 1 Department of Chemistry, Imperial College London, London, W12 0BZ, UK; 2 Centre for Processable Electronics, Imperial College London, London, W12 0BZ, UK; 3 Department of Chemistry, Loughborough University, Loughborough, LE11 3TU, UK

**Keywords:** macrocycles, π-conjugated macrocycles, paracyclophanetetraene, PCT, poly(p-phenylene vinylene), PPV, visualisation of chemical shielding tensors, VIST, aromaticity, antiaromaticity, rotamers, Wittig reaction, photoluminescence, cyclic voltammery

## Abstract

**Background**: Poly( *p*-phenylene vinylene)s ( **PPV**s) and [2.2.2.2]paracyclophanetetraene ( **PCT**) are both composed of alternating π-conjugated *para*-phenylene and vinylene units. However, while the former constitute a class of π-conjugated polymers that has been used in organic electronics for decades, the latter is a macrocycle that only recently revealed its potential for applications such as organic battery electrodes. The cyclic structure endows **PCT** with unusual properties, and further tuning of these may be required for specific applications. **Methods**: In this article, we adopt an approach often used for tuning the properties of **PPV**s, the introduction of alkoxy (or alkylthio) substituents at the phenylene units, for tuning the optoelectronic properties of **PCT**. The resulting methoxy- and methylthio-substituted **PCT**s, obtained by Wittig cyclisation reactions, are studied by UV-vis absorption, photoluminescence, and cyclic voltammetry measurements, and investigated computationally using the visualisation of chemical shielding tensors (VIST) method. **Results**: The measurements show that substitution leads to slight changes in terms of absorption/emission energies and redox potentials while having a pronounced effect on the photoluminescence intensity. The computations show the effect of the substituents on the ring currents and chemical shielding and on the associated local and global (anti)aromaticity of the macrocycles, highlighting the interplay of local and global aromaticity in various electronic states. **Conclusions**: The study offers interesting insights into the tuneability of the properties of this versatile class of π-conjugated macrocycles.

## Introduction

Poly( *p*-phenylene vinylene)s ( **PPV**s) are among the best investigated π-conjugated polymers ^1, 2^. They are composed of alternating *para*-phenylene and vinylene units and usually feature substituents attached to the π-conjugated backbone to modify their optoelectronic and dissolution properties. Most frequently, alkoxy substituents are attached to the phenylene units, but the corresponding alkylthio-substituted **PPV**s have also been reported ^3^. The alkyl part of the substituents (often a linear or branched alkyl chain) is intended to improve the solubility, while the purpose of the oxygen or sulfur atom linking the alkyl part to the backbone is to modify the optoelectronic properties, mainly through their positive mesomeric effect.

**Figure 1.**
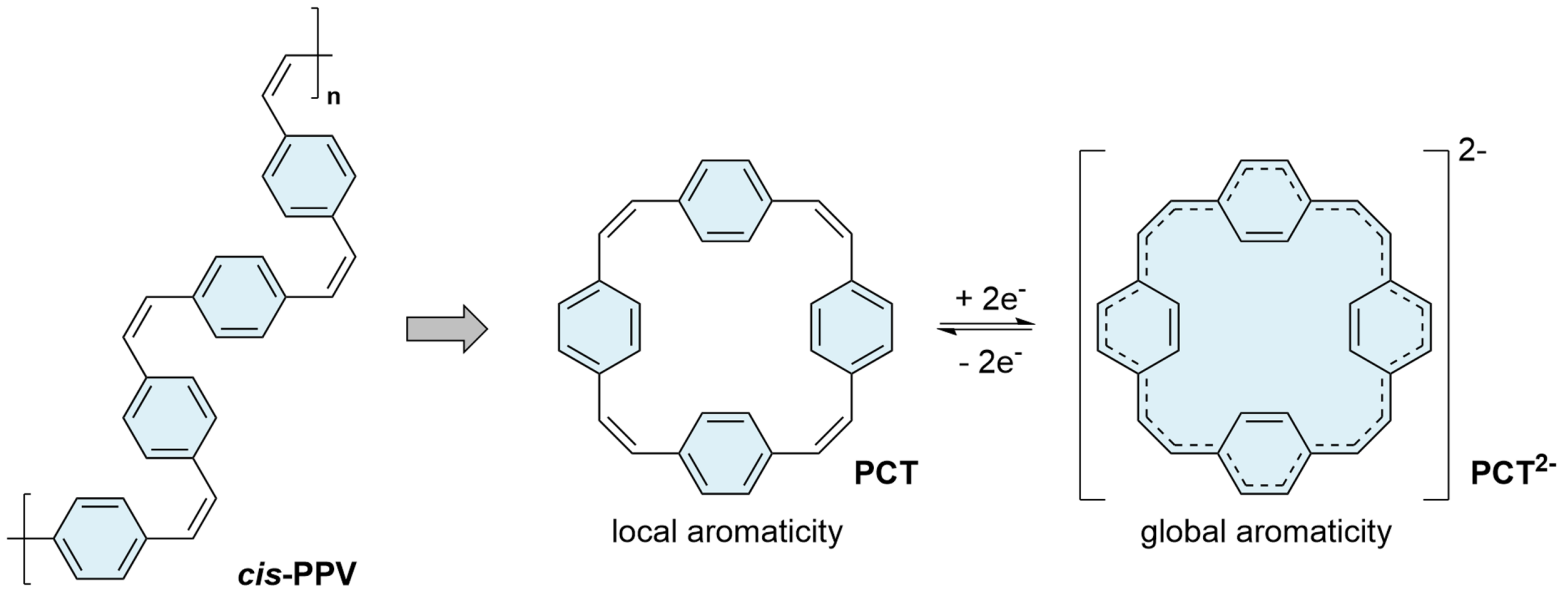
[2.2.2.2]Paracyclophanetetraene ( **PCT**) as a cyclic structural analogue of *cis*-poly( *p*-phenylene vinylene) ( *cis*- **PPV**) (left); reversible two-electron reduction and aromaticity switching of **PCT** (right).

[2.2.2.2]Paracyclophanetetraene ( **PCT**) can be seen as a cyclic structural analogue of unsubstituted **PPV** ( Figure 1). This π-conjugated macrocycle, first synthesized in the 1970s ^4^, was recently rediscovered by us and has proven to be a capable battery electrode material ^5^. The excellent redox and charge storage properties of **PCT** have been attributed to ring currents and voids enabled by the cyclic structure of the molecule, suggesting that the optoelectronic properties of **PCT** and **PPV** differ significantly, despite their structural similarity. In contrast to the polymers, the vinylene units in **PCT** must adopt a *cis*-configuration; no end groups are present that may affect the properties.

The aim of the work presented here was to synthesize and study **PCT** derivatives with alkoxy and alkylthio substituents at the phenylene units, in analogy to the well-investigated substituted **PPV**s. In contrast to **PPV**, **PCT** dissolves well in organic solvents also without substituents with linear or branched alkyl chains. Thus, simple methyl groups were selected as the alkyl part of the substituents. When studying the properties of the resulting methoxy- and methylthio-substituted **PCT** derivatives, we were particularly interested in the effect of the substituents on the ring currents, having recently reported the drastic effects of the introduction of ester groups (at the vinylene units) on these currents ^6^.

## Results and discussion

### Synthesis

**Figure 2.**
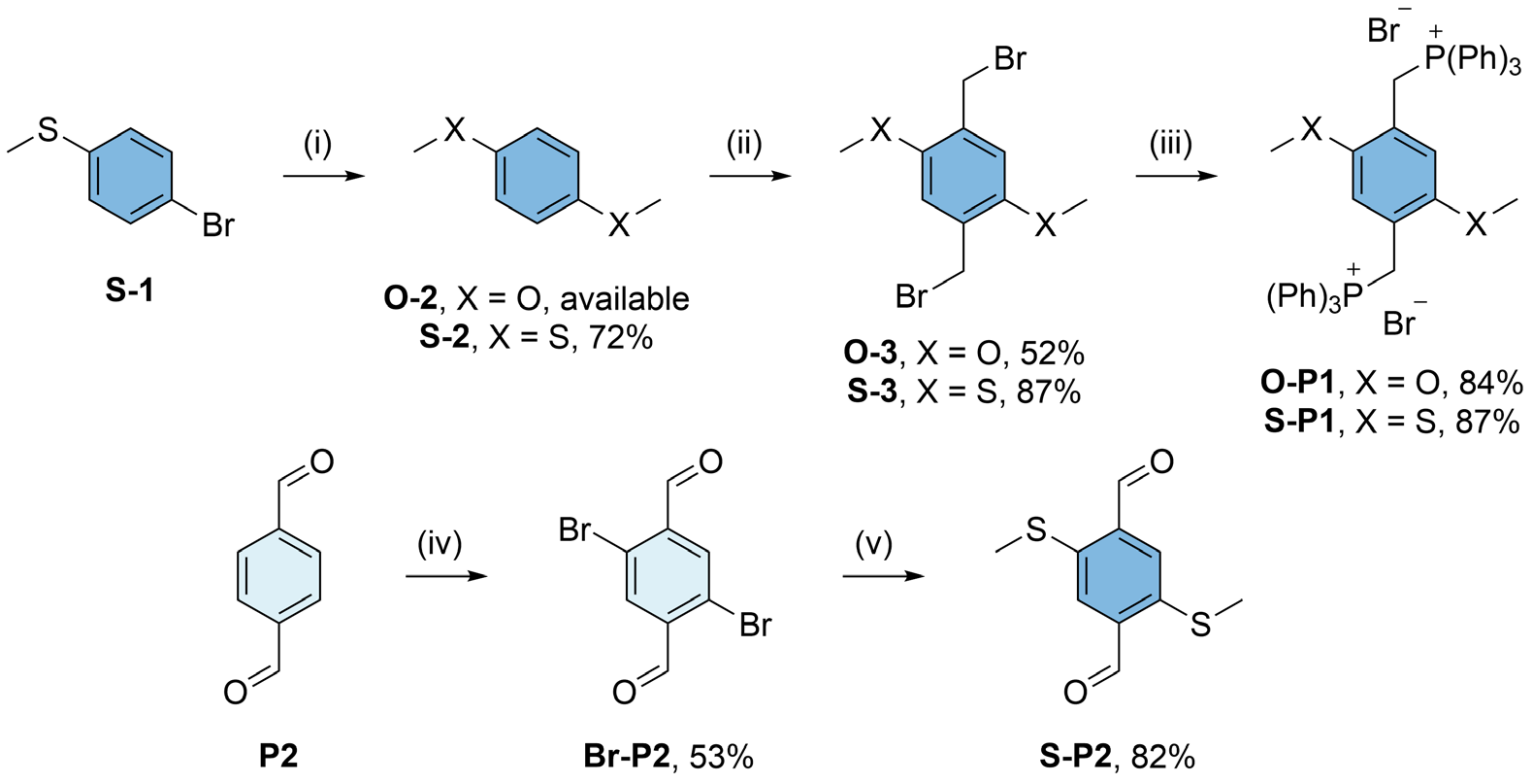
Synthesis of methoxy- and methylthio-substituted Wittig cyclisation precursors **O-P1**, **S-P1**, and **S-P2**. Reaction conditions: (i) CuI, Cu(OAc) _2_, dimethyl sulfoxide (DMSO), 135 °C; (ii) paraformaldehyde, HBr in acetic acid, 1,4-dioxane (X = O) / formic acid (X = S), 80 °C; (iii) PPh _3_, toluene, 120 °C; (iv) N-bromosuccinimide (NBS), conc. H _2_SO _4_, 60 °C; (v) NaSCH _3_, dimethylformamide (DMF), r.t..

Unsubstituted **PCT** can be synthesized by a Wittig cyclisation reaction in yields of 10% to 15% using [1,4-phenylenebis(methylene)]bis(triphenylphosphonium) dibromide ( **P1**) and terephthalaldehyde ( **P2**) as the cyclisation precursors ^4, 5^. Aiming for an analogous synthesis of methoxy- and methylthio-substituted **PCT** derivatives, we first synthesized the substituted Wittig cyclisation precursors **O-P1**, **S-P1**, and **S-P2** ( Figure 2): The phosphonium salts **O-P1** and **S-P1** were synthesized via the same route, but while 1,4-dimethoxybenzene ( **O-2**) for the synthesis of **O-P1** is readily available from commercial suppliers, the corresponding methylthio-substituted compound 1,4-bis(methylthio)benzene ( **S-2**) for the synthesis of **S-P1** was obtained by a copper-catalysed conversion of 4-bromothioanisole ( **S-1**) in dimethyl sulfoxide (DMSO) ^7^. Compounds **O-2** and **S-2** were then bromomethylated by adapting our previously reported procedures to these substrates ^3^. The reactions yielded compounds **O-3** and **S-3** in yields of 52% and 87%, respectively. In the final step, **O-3** and **S-3** were reacted with triphenylphosphine (PPh _3_) in boiling toluene, affording **O-P1** and **S-P1** as white solids in good yields. In contrast, the dialdehyde **S-P2** was synthesized by slightly adapting published procedures for the bromination of **P2** in the first step and nucleophilic aromatic substitution with sodium methanethiolate (NaSCH _3_) in the second step ^8, 9^.

**Figure 3.**
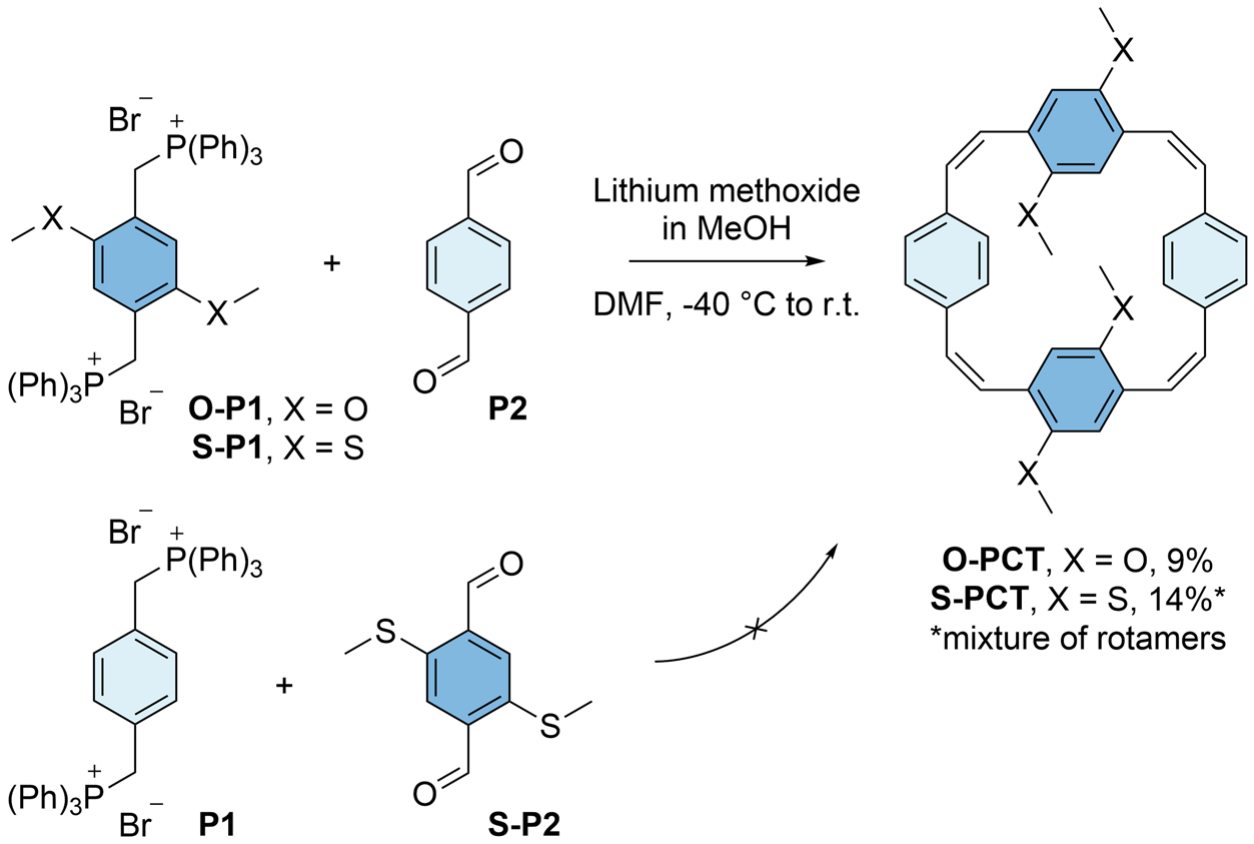
Synthesis of methoxy- and methylthio-substituted [2.2.2.2]paracyclophanetetraenes **O-PCT** and **S-PCT** by Wittig cyclisation reactions of precursors **O-P1**/ **S-P1** and **P2** (top). The analogous reaction of precursors **P1** and **S-P2** did not yield the product (bottom).

The phosphonium salts **O-P1** and **S-P1** were then reacted with terephthalaldehyde ( **P2**) in dimethylformamide (DMF) at a low temperature of -40 °C ( Figure 3). As for the synthesis of **PCT** ^5^, lithium methoxide dissolved in anhydrous methanol (MeOH) was used as the base for these Wittig cyclisation reactions. The base was added slowly using a syringe pump, affording the macrocycles **O-PCT** and **S-PCT** in yields of 9% and 14%, respectively, after work-up and purification by gel permeation chromatography (GPC). In contrast, placing the methylthio substituents on the aldehyde precursor instead of the phosphonium salt, reacting *p*-xylylenebis(triphenylphosphonium bromide) ( **P1**) with precursor **S-P2** under the same reaction conditions, did not afford any **S-PCT**, presumably due to a less favourable *cis*/ *trans* ratio of the vinylene units formed in this Wittig reaction (the electronic character of the precursors can influence the ratio). The reaction of **S-P1** and **S-P2** to obtain the macrocycle with substituents on all four phenylene units has also been attempted but did not give the desired product.

### Conformation

**Figure 4.**
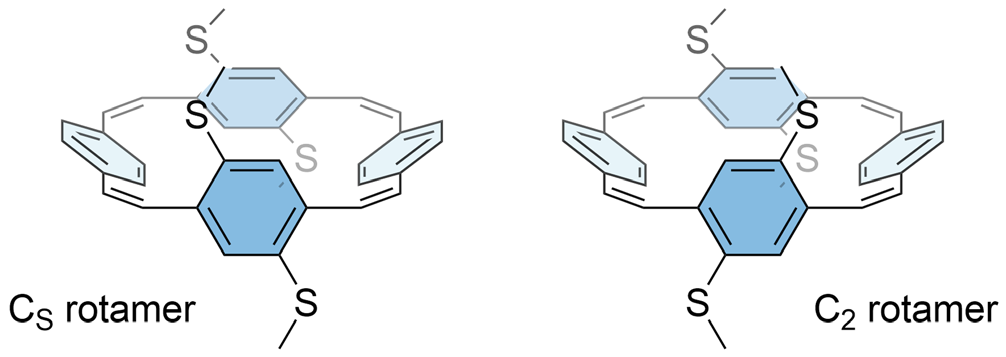
Illustration of the two rotamers of **S-PCT**, classified according to their idealised symmetry properties. The computed energy barrier for interconversion is 94 kJ/mol.

^1^H NMR spectra of **S-PCT** recorded at room temperature and 50 °C indicated the presence of two rotamers that could not be separated (see Figure 4 for an illustration of the two rotamers). A rotamer ratio of approximately 10:1, which corresponds to a free energy difference of about 6 kJ/mol, was determined from the integrals. In contrast, the ^1^H NMR spectrum of **O-PCT** did not show the presence of rotamers, indicating a lower energy barrier for the rotation of the phenylene units with methoxy substituents than with methylthio substituents.

To test these assumptions, computations were carried out to estimate the energy barrier for the interconversion of the two rotamers. Indeed, a barrier of only 45 kJ/mol was found for **O-PCT**. According to the Eyring equation, this corresponds to an interconversion time of about 10 µs, which is well below the time scale relevant to NMR. Both rotamers are very similar in energy, with the C _s_ rotamer 3.2 kJ/mol below the C _2_ rotamer, suggesting that both are present at room temperature. **S-PCT**, on the other hand, showed a significantly enhanced barrier of 94 kJ/mol with an associated interconversion time well above one hour. The C _2_ rotamer was found to be more stable by 16.6 kJ/mol, which is similar but somewhat larger than the free energy difference deduced from experiment (see above). The difference in behaviour between the two molecules can be understood by the fact that the methylthio groups are bulkier, causing steric strain for the transition state and the C _s_ rotamer. Taking the neutral C _2_ rotamer as an example, we find that the two C-O bonds in **O-PCT** are 1.35 and 1.39 Å in length whereas the C-S bonds in **S-PCT** measure 1.77 and 1.81 Å.

### UV-vis absorption and photoluminescence

**Figure 5.**
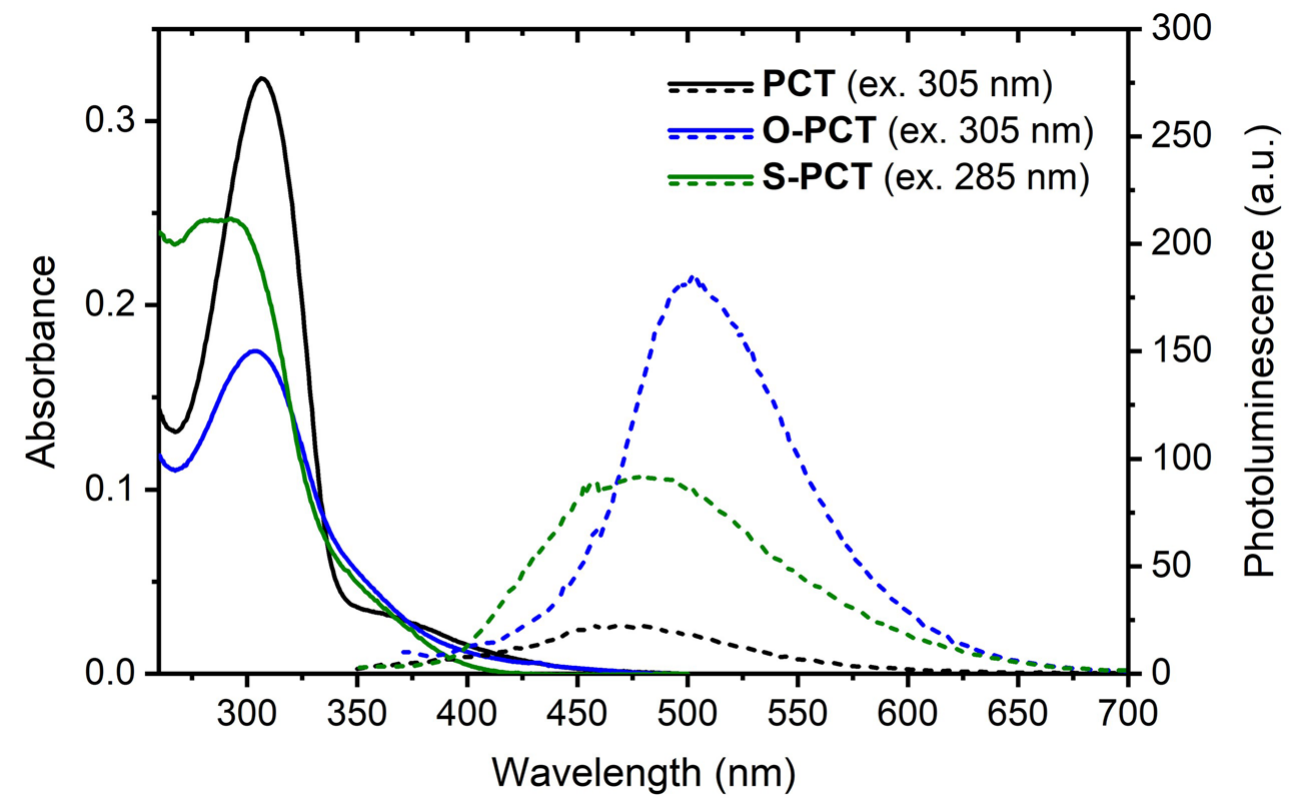
UV-vis absorption (solid lines) and photoluminescence spectra (dashed lines) of the macrocycles in CHCl _3_ solution (approx. 5 μM). The excitation wavelengths for recording the photoluminescence (PL) spectra are shown in brackets.

UV-vis absorption measurements in CHCl _3_ solution ( Figure 5, solid lines) showed slightly blueshifted absorption maxima for **O-PCT** (λ _abs,max_ = 304 nm) and **S-PCT** (λ _abs,max_ = 293 nm) compared to **PCT** (λ _abs,max_ = 306 nm). The second absorption peak in the spectrum of **S-PCT** (λ _abs_ = 283 nm) may be attributed to the presence of two rotamers. Photoluminescence (PL) measurements of the solutions ( Figure 5, dashed lines) showed a significant increase in PL intensity upon introduction of the substituents, particularly upon introduction of the methoxy substituents. Similar sensitivity to the substitution pattern for the PL intensity were also found for the related ester-substituted molecules and can be tentatively assigned to symmetry breaking in the excited state, lifting the selection rules for the formally symmetry-forbidden S _1_ state ^6^, see, e.g., reference 10 for a discussion of the underlying physics. In contrast to the absorption maxima, the PL maxima of **O-PCT** (λ _PL,max_ = 502 nm) and **S-PCT** (λ _PL,max_ = 481 nm) were found to be redshifted compared to **PCT** (λ _PL,max_ = 468 nm), thus increasing their Stokes shifts from 1.40 eV for **PCT** to 1.61/1.65 eV for **O-PCT**/ **S-PCT**. It is worth noting that the Stokes shifts of the linear **PCT** analogue containing 4 phenylene-vinylene units (λ _abs,max_ = 396 nm; λ _PL,max_ = 463 nm) ^11^, of alkoxy-substituted **PPV** (λ _abs,max_ = 502 nm; λ _PL,max_ = 558 nm) ^12^, and alkylthio-substituted **PPV** (λ _abs,max_ = 453 nm; λ _PL,max_ = 527 nm) ^3^ are all well below 0.5 eV, highlighting the dramatic effect of the cyclic conjugation. Indeed, the large Stokes shifts of the substituted and unsubstituted **PCT**s, resulting in almost no spectral overlap between absorption and emission, can be seen as a signature of excited-state aromaticity, *cf.* references 6 and 13. Thus, the spectra suggest that symmetry breaking is only a dynamic effect accounting for a minor perturbation of excited-state aromaticity (see below for a further discussion).

### Redox potentials

**Figure 6.**
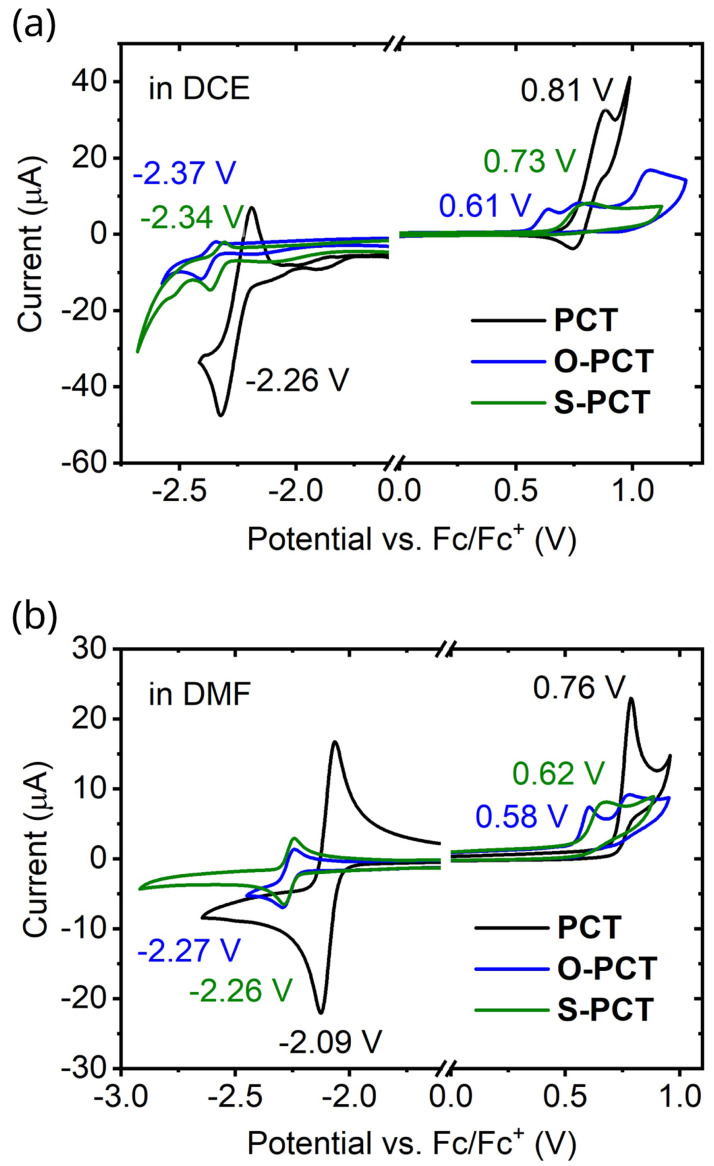
Cyclic voltammograms of the macrocycles in 1,2-dichloroethane (DCE) and dimethylformamide (DMF) recorded using a glassy carbon working electrode, a platinum mesh auxiliary electrode, and a silver wire quasi-reference electrode (QRE) at a scan rate of 0.1 V s ^-1^. 0.1 M tetrabutylammonium hexafluorophosphate (NBu _4_PF _6_) was used as the supporting electrolyte.

Cyclic voltammetry measurements of the macrocycles in 1,2-dichloroethane (DCE) and dimethylformamide (DMF) ( Figure 6) were carried out to determine the redox potentials *vs.* ferrocene/ferrocene ^+^ (Fc/Fc ^+^) in two different solvents. As a general trend, the redox potentials for both the reductions and oxidations shifted to lower values upon introduction of the substituents, with a slightly larger shift observed for the introduction of the methoxy substituents. This can be explained by the electron-donating character of the substituents.

In DCE, the redox potential for the first reduction wave shifted from -2.26 V for **PCT** to -2.34 V for **S-PCT** and -2.37 V for **O-PCT**. For **S-PCT**, a second reduction wave was observed at -2.50 V. The redox potential for the first oxidation wave shifted from 0.81 V for **PCT** to 0.73 V for **S-PCT** and 0.61 V for **O-PCT**. For **S-PCT**, the first derivative of the measurement indicated further oxidations at 0.79 V and 1.08 V. For **O-PCT**, a second and third oxidation wave were observed at 0.73 V and 1.03 V, respectively.

In DMF, the redox potential for the first reduction wave showed a shift from -2.09 V for **PCT** to -2.26 V for **S-PCT** and -2.27 V for **O-PCT**. No further reduction waves were observed in this solvent. The redox potential for the first oxidation wave shifted from 0.76 V for **PCT** to 0.62 V for **S-PCT** and 0.58 V for **O-PCT**. A second oxidation wave was observed at 0.83 V for **S-PCT** and 0.74 V for **O-PCT**.

Computations place the potentials for concerted two-electron reduction in DCE at -2.28, -2.40, and -2.35 V for **PCT**, **O-PCT**, and **S-PCT**, respectively, suggesting that the experimental reduction potentials refer to two-electron processes. Potentials for two-electron oxidation are placed at 0.89, 0.75, and 0.98 V for the three molecules. Considering that the values for **O-PCT** and **S-PCT** are considerably higher than the measured first oxidation potentials, this suggests that oxidation proceeds via one-electron processes.

### Ring currents and chemical shielding

To study the effect of the methoxy and methylthio substituents on the magnetic properties (ring currents and chemical shielding) and on the associated local and global (anti)aromaticity of the macrocycles, the visualisation of chemical shielding tensors (VIST) method ^14^, which is based on the nucleus independent chemical shift ^15^, was used. As explained previously ^6, 14^, VIST allows the visualisation of local variations in aromaticity and antiaromaticity in the context of the molecular structure by showing the chemical shielding tensor components using a representation of blue (shielded, aromatic) or red (deshielded, antiaromatic) dumbbells. Each tensor component relates to ring currents in a plane perpendicular to it.

**Figure 7.**
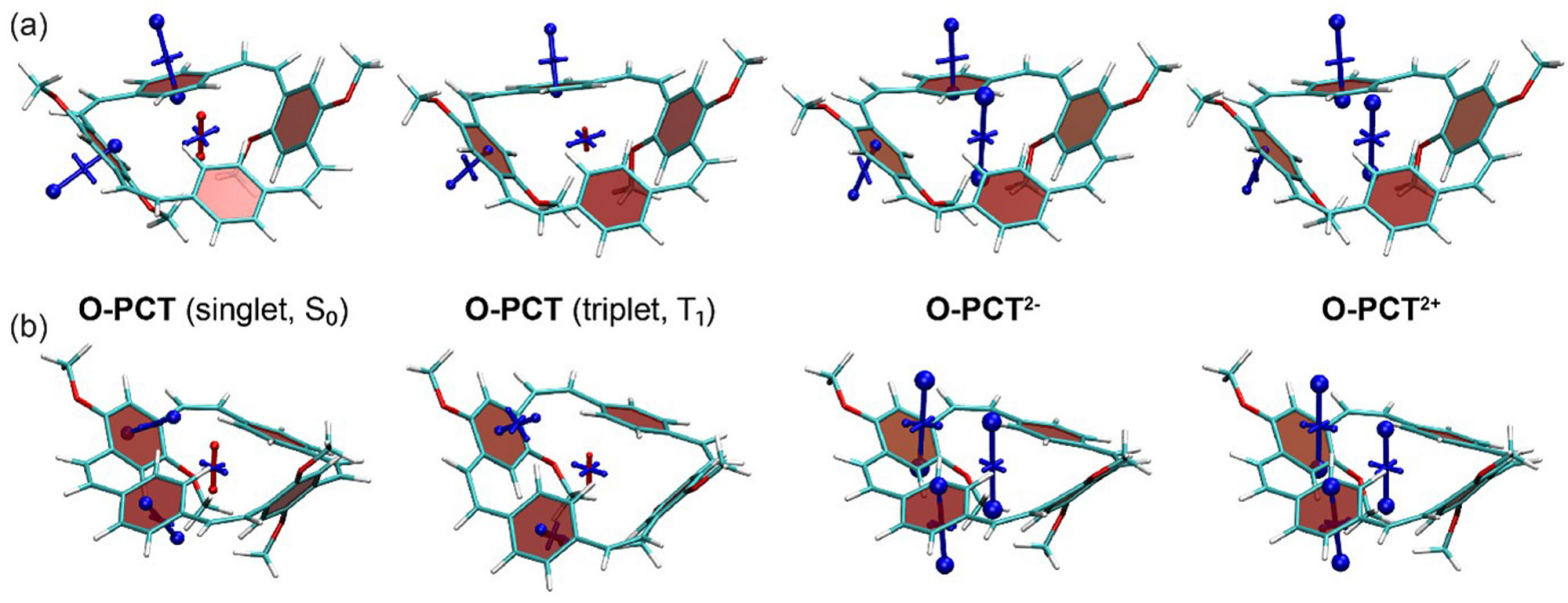
VIST plots for the rotamers ( **a**) C _2_ and ( **b**) C _s_ of **O-PCT** (classified according to their idealised symmetry properties) in different charge and spin states. Shielded (aromatic) tensor components are shown in blue, deshielded (antiaromatic) tensor components in red. Each tensor component relates to ring currents in a plane perpendicular to it.

**Figure 8.**
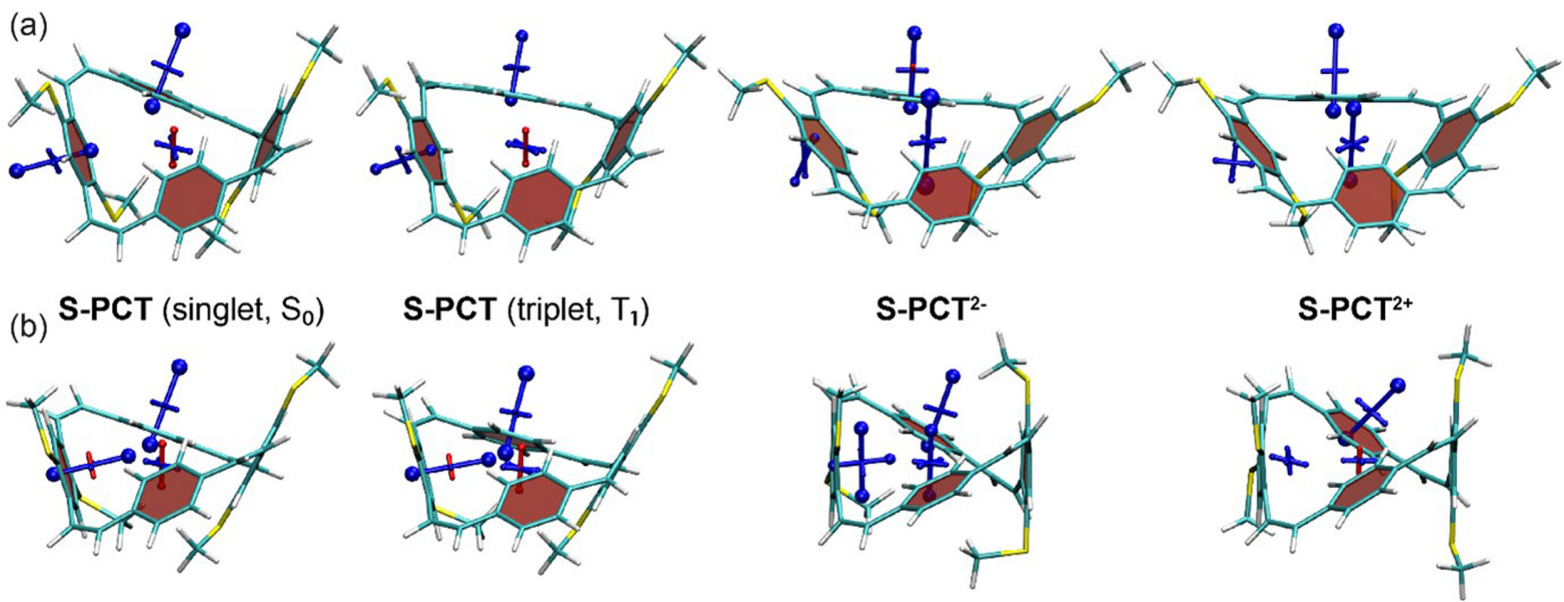
VIST plots for the rotamers ( **a**) C _2_ and ( **b**) C _s_ of **S-PCT** (classified according to their idealised symmetry properties) in different charge and spin states. Shielded (aromatic) tensor components are shown in blue, deshielded (antiaromatic) tensor components in red. Each tensor component relates to ring currents in a plane perpendicular to it.

The VIST plots of **O-PCT** and **S-PCT** in the neutral S _0_ and T _1_ states as well as in the doubly charged S _0_ states are provided in Figure 7 and Figure 8, respectively. Shielding tensors were computed at the centre of the ring to probe global ring currents as well as 1 Å off the centre of the phenylene units to probe their local aromaticity. Both rotamers of the macrocycles were analysed. The corresponding VIST plots of **PCT** are provided in our previous work ^6^. The substituents are seen to have little effect on the magnetic properties of the neutral S _0_ state. As in our previous analysis of **PCT**, the main component of the chemical shielding tensors located 1 Å off the planes of the phenylene units is shielded and almost perpendicular to the planes, indicating their local aromaticity. At the centre of the macrocycle, the tensor component perpendicular to the plane of the macrocycle is slightly deshielded, possibly indicating weak global antiaromaticity of the macrocyclic [4n] π-electron system.

**Figure 9.**
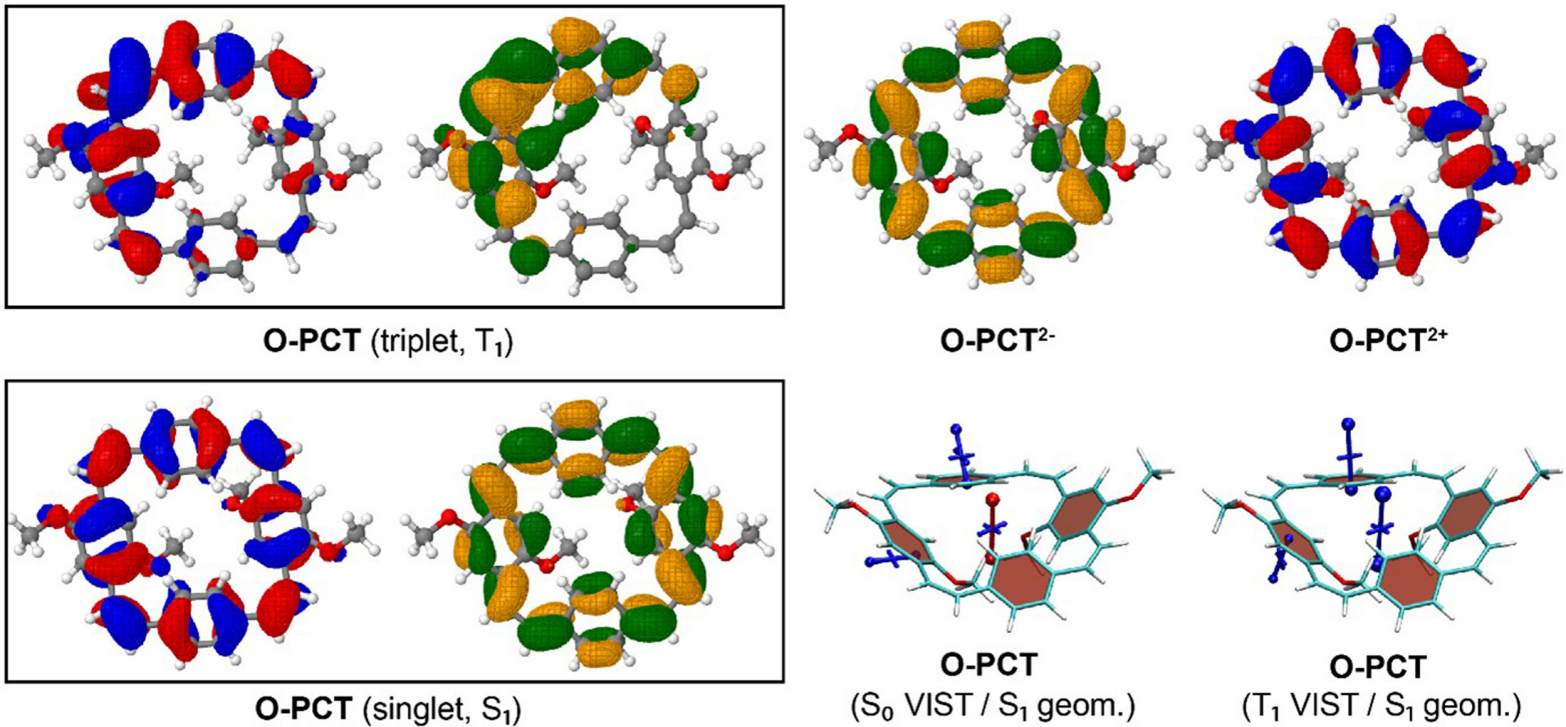
Dominant natural difference orbitals (NDOs) (blue/red for electron detachment; green/orange for attachment) for different electronic states of **O-PCT** (C _2_ rotamer) and VIST plots for the S _0_ and T _1_ states at the S _1_ geometry.

In contrast to the S _0_ state, the magnetic properties of the neutral T _1_ state of **O-PCT** and **S-PCT** differ significantly from those of **PCT**. The VIST plots of **PCT** in the T _1_ state indicate strong aromatic macrocyclic currents (and perturbed local aromaticity). This macrocyclic Baird aromaticity is obliterated by the introduction of the substituents; as the S _0_ states, the neutral T _1_ states of **O-PCT** and **S-PCT** are dominated by the local aromaticity of the phenylene units. To explain this phenomenon, we analysed the electronic structure in more detail by computing the natural difference orbitals (NDOs) ^16^ between the S _0_ and T _1_ states of the C _2_ rotamer of **O-PCT** as an example. The NDOs, shown on the upper left in Figure 9, reveal that in the T _1_ state the symmetry is broken, and the excitation is localised on one side of the molecule. More specifically, the excitation is centred around one of the vinyl groups, which is strongly twisted out of plane. Following the arguments in Ref. 17, it can be understood that Baird aromaticity would only be achieved if the transition occurred between delocalised orbitals in a cyclically conjugated structure. Next, we were interested whether similar symmetry breaking occurs for the S _1_ state. Therefore, the S _1_ state was optimised, and the NDOs obtained are shown on the lower left in Figure 9. In contrast to the T _1_ state, the excitation in the S _1_ state is evenly delocalised over the whole macrocycle. Closer inspection shows that both NDOs possess 12 nodal planes, corresponding to the quasidegenerate HOMO and LUMO of the parent [24]annulene structure. The excitation occurs between these quasidegenerate orbitals, which is the signature of excited-state aromaticity within the MO picture ^17^ and is, thus, consistent with the large Stokes shift observed in the measurements. The computation of shielding tensors in the S _1_ state is not routinely possible. Therefore, we have computed VIST plots of the S _0_ and T _1_ states at the S _1_ geometry instead ( Figure 9, bottom right). These show enhanced antiaromaticity and aromaticity compared to the VIST plots at the geometries optimized for the respective states, further confirming that the S _1_ geometry facilitates delocalisation.

Differently to the T _1_ state, the VIST plots of the doubly charged states of **O-PCT** and **S-PCT** ( Figure 7 and Figure 8) indicate similarly strong macrocyclic aromaticity as observed for **PCT ^2-^** and **PCT ^2+^** with the exception of the C _s_ rotamer of **S-PCT ^2+^** for which the VIST plot does not indicate any macrocyclic currents. In all other cases, the main component of the chemical shielding tensors at the phenylene units are tilted and almost perpendicular to the plane of the macrocycle, indicating strong perturbation of the local aromaticity by macrocyclic currents. The central shielding tensors also indicate the strong macrocyclic currents. To exemplify the change in electronic structure, we present the NDOs of the C _2_ rotamer of **O-PCT** ( Figure 9, upper right). The relevant attachment NDO for the dianion as well as the detachment NDO for the dication are both evenly delocalised over the entire macrocycle. More specifically, they are of similar shape as the S _1_ NDOs, possessing the 12 nodal planes corresponding to the quasidegenerate [24]annulene frontier orbitals. Revisiting the C _s_ rotamer of **S-PCT ^2+^**, we find that its relevant orbitals do not possess the required cyclically delocalised structure, thus explaining the lack of aromaticity.

In summary, we find that **O-PCT** and **S-PCT** possess similar electronic structure as the parent **PCT** molecule. But similarly to our previously discussed ester-substituted molecules ^6^, we find that due to their lower symmetry they are further removed from the underlying idealised antiaromatic [24]annulene structure. The large Stokes shifts seen in the UV/vis absorption and PL spectra ( Figure 5) are consistent with excited-state aromaticity, as seen for the optimised S _1_ state of **O-PCT** in Figure 9. On the other hand, we tentatively assign the enhanced photoluminescence activity of the substituted molecules to an increased propensity for symmetry breaking, which is seen explicitly in the T _1_ optimised structure and probably also plays a role for the S _1_ state in terms of structural fluctuations. However, a full ab initio simulation of the resulting spectra is out of the scope of this work.

## Conclusions

Our study shows that methoxy- and methylthio-substituted **PCT** derivatives can be obtained by Wittig cyclisation reactions of substituted [1,4-phenylenebis(methylene)]bis(triphenylphosphonium) dibromides and terephthalaldehyde, in similar yields as reported for the synthesis of unsubstituted **PCT**. The required substituted Wittig cyclisation precursors can be obtained in good yields via a two/three-step procedure. Placing substituents on the terephthalaldehyde precursor did not yield the macrocycles in our attempts.

While the energy barrier for the rotation of the methoxy-substituted phenylene units in **O-PCT** was found to allow a rapid interconversion of the two rotamers, the significantly higher energy barrier for the rotation of the methylthio-substituted phenylene units in **S-PCT** resulted in distinct ^1^H NMR signals that can be assigned to the two rotamers. Computations supported this finding, highlighting that the interconversion barrier in **S-PCT** is more than twice the barrier in **O-PCT**, increasing the expected interconversion time from 10 µs to well over one hour. The larger barrier was related to the bulkier nature of the methylthio substituents when compared to the methoxy substituents.

The introduction of the substituents was also found to alter the optoelectronic properties of **PCT**. In particular, the maximum photoluminescence (PL) intensity of **O-PCT** was found to be approximately eight times higher than for **PCT**, a feature that we assign to dynamic excited-state symmetry breaking and the associated lifting of selection rules. While the PL maxima were found to be redshifted, the UV-vis absorption maxima of **O-PCT** and **S-PCT** were slightly blueshifted compared to **PCT**, thus increasing the overall Stokes shifts to above 1.5 eV, which is more than three times the value of the linear **PPV** analogues. Furthermore, the electron-donating character of the substituents leads to a shift of the redox potentials to lower values, as confirmed by cyclic voltammetry measurements in two different solvents along with computations. This can be an interesting feature for tuning the properties for applications such as organic battery electrodes and further confirms the high tuneability of the properties of this compound class.

In the neutral and most of the doubly charged singlet states of **O-PCT** and **S-PCT**, the ring currents and chemical shielding do not differ significantly from those of **PCT** in the same states, according to our computational investigation using the visualisation of chemical shielding tensors (VIST) method. However, in the neutral T _1_ state, the macrocyclic Baird aromaticity observed in the VIST plots of **PCT** is obliterated by the introduction of the substituents and the associated breaking of the symmetry; the molecules are dominated by the local aromaticity of the phenylene units.

In summary, the study expands the set of available [2.2.2.2]cyclophanetetraenes and offers further insights into the tuneability of the properties of this versatile compound class.

## Methods

### Synthetic methods

Reagents and solvents for the synthesis were purchased from commercial suppliers and used without further purification, including compounds **S-1** (Sigma-Aldrich, product number 196525), **O-2** (Sigma-Aldrilch, product number D131350), **P1** (Alfa Aesar, product number A18241.14), and **P2** (Sigma-Aldrich, product number 808617). Purification by recycling preparative GPC was carried out on a LaboACE LC-5060 (Japan Analytical Industry Co., Tokyo, JAPAN) system equipped with a JAIGEL-2HR column and a TOYDAD800-S detector.

**1,4-Bis(methylthio)benzene (S-2):** Synthesis adapting a published procedure ^7^. 4-Bromothioanisole ( **S 1**) (508 mg, 2.5 mmol, 1.0 equiv.), CuI (119 mg, 0.63 mmol, 0.25 equiv.) and Cu(OAc) _2_ (908 mg, 5.0 mmol, 2.0 equiv.) were added to an oven-dried vial, purged with nitrogen and sealed before adding 8 mL DMSO. The suspension was heated to 135 °C for 36 hours before cooling to room temperature (r.t.) and adding 40 mL Et _2_O. The orange suspension was filtered off and washed with cold H _2_O before extracting the filtrate with Et _2_O (3x), drying the organic phase with Na _2_SO _4_ and removing the solvent under reduced pressure. Product **S-2** was purified by column chromatography using hexane/Et _2_O (10:1), yielding an off-white, wax-like solid (305 mg, 1.8 mmol, 72%). ^1^H NMR (400 MHz, CDCl _3_): δ 7.20 (s, 4H), 2.47 (s, 6H) ppm; in accordance with the literature ^7^.

**2,5-Bis(bromomethyl)-1,4-dimethoxybenzene (O-3):** Synthesis adapting a procedure used for the bromomethylation of 1,4-dihexyloxybenzene ^3^. 1,4-Dimethoxybenzene ( **O-2**) (691 mg, 5.0 mmol, 1.0 equiv.) and paraformaldehyde (901 mg, 30 mmol, 6.0 equiv.) were suspended in 20 mL 1,4-dioxane. 8.0 mL HBr (30% in acetic acid) was added dropwise and the reaction was heated to 80 °C for 24 hours. The white suspension was slowly cooled to r.t., allowing the product to crystallise as a white solid. The solid was filtered off, washed twice with H _2_O and recrystallised from acetonitrile (MeCN), yielding product **O-3** as white needles (837 mg, 2.6 mmol, 52%). ^1^H NMR (400 MHz, CDCl _3_): δ 6.87 (s, 2H), 4.54 (s, 4H), 3.87 (s, 6H) ppm; in accordance with the literature ^18^.

**2,5-Bis(bromomethyl)-1,4-bis(methylthio)benzene (S-3):** Synthesis adapting a procedure used for the bromomethylation of related substrates ^3^. 1,4-Bis(methylthio)benzene ( **S-2**) (375 mg, 2.2 mmol, 1.0 equiv.) and paraformaldehyde (264 mg, 8.8 mmol, 4.0 equiv.) were dissolved in 20 mL formic acid while heating to 80 °C. 3.1 mL HBr (30% in acetic acid) were added and the reaction was stirred at 80 °C for 1 hour. Paraformaldehyde (264 mg, 8.8 mmol, 4.0 equiv.) and 3.1 mL HBr (30% in acetic acid) were added three more times in intervals of 1 hour; the reaction was then stirred at 80 °C overnight using a magnetic stirrer. The white suspension was slowly cooled to r.t., allowing the product to crystallise as an off-white solid. Product **S-3** was isolated by filtering and washing with MeOH (685 mg, 1.9 mmol, 87%). ^1^H NMR (400 MHz, CD _2_Cl _2_): δ 7.28 (s, 2H), 4.63 (s, 4H), 2.52 (s, 6H) ppm. ^1^H NMR (400 MHz, CDCl _3_): δ 7.26 (s, 2H, overlaps with solvent peak), 4.61 (s, 4H), 2.52 (s, 6H) ppm; ^13^C{ ^1^H} NMR (101 MHz, CDCl _3_): δ 137.2, 136.0, 129.5, 31.1, 16.7 ppm. HRMS ( *m/z*): [M] ^+^ calcd for C _10_H _12_S _2_Br _2_: 353.8742, found: 353.8733 (APCI).

**[(2,5-dimethoxy-1,4-phenylene)bis(methylene)]bis(triphenylphosphonium) dibromide (O-P1):** Substrate **O-3** (713 mg, 2.2 mmol, 1.0 equiv.) and PPh _3_ (2.02 g, 7.7 mmol, 3.5 equiv.) were suspended in 7.5 mL anhydrous toluene in a sealed vial under nitrogen and heated to 120 °C overnight. The white precipitate was filtered and washed with toluene and Et _2_O. Product **O-P1** was isolated as a white solid (1.56 g, 1.8 mmol, 84%). ^1^H NMR (400 MHz, CDCl _3_): δ 7.79 – 7.73 (m, 6H), 7.73 – 7.61 (m, 24H), 6.96 (d, *J* = 1.9 Hz, 2H), 5.23 (d, *J* = 12.8 Hz, 4H), 2.97 (s, 6H) ppm. ^31^P{ ^1^H} NMR (162 MHz, CDCl _3_): δ 21.82 ppm. HRMS ( *m/z*): [M–2Br] ^2+^ calcd for C _46_H _42_Br _2_O _2_P _2_: 344.1330, found: 344.1325 (ESI).

**[(2,5-bis(methylthio)-1,4-phenylene)bis(methylene)]bis(triphenylphosphonium) dibromide (S-P1):** Substrate **S-3** (605 mg, 1.7 mmol, 1.0 equiv.) and PPh _3_ (1.34 g, 5.1 mmol, 3.0 equiv.) were suspended in 25 mL anhydrous toluene in a sealed vial under nitrogen and heated to 120 °C overnight. The white precipitate was filtered and washed with toluene and Et _2_O. Product **S-P1** was isolated as a white solid (1.30 g, 1.5 mmol, 87%) after triturating in boiling *n*-butanol for 1.5 h for further purification. ^1^H NMR (400 MHz, CDCl _3_): δ 7.82 – 7.74 (m, 6H), 7.73 – 7.59 (m, 24H), 7.13 (d, *J* = 2.1 Hz, 2H), 5.52 (d, *J* = 12.5 Hz, 4H), 1.71 (s, 6H) ppm. ^31^P{ ^1^H} NMR (162 MHz, CDCl _3_): δ 22.00 ppm. HRMS ( *m/z*): [M–2Br] ^2+^ calcd for C _46_H _42_Br _2_P _2_S _2_: 360.1101, found: 360.1111 (ESI).

**2,5-Dibromoterephthalaldehyde (Br-P2):** Synthesis adapting a published procedure ^8^. Terephthalaldehyde ( **P2**) (8.05 g, 60 mmol, 1.0 equiv.) was dissolved in 80 mL conc. H _2_SO _4_ at 60 °C. N-Bromosuccinimide (NBS) (23.5 g, 132 mmol, 2.2 equiv.) was added in small portions over 30 min. The reaction was then stirred for 3 hours at 60 °C before cooling to r.t. and pouring onto ice. The white precipitate was washed with aq. NaHCO _3_ and brine before being recrystallised from CHCl _3_. **Br-P2** was isolated as off-white crystals (9.30 g, 31.9 mmol, 53%). ^1^H NMR (400 MHz, CDCl _3_): δ 10.35 (s, 2H), 8.16 (s, 2H) ppm; in accordance with the literature ^19^.

**2,5-Bis(methylthio)terephthalaldehyde (S-P2):** Synthesis adapting a published procedure ^9^. **Br-P2** (992 mg, 3.4 mmol, 1.0 equiv.) was dissolved in 70 mL DMF before adding NaSCH _3_ (498 mg, 7.1 mmol, 2.1 equiv.) at r.t. The dark red solution was stirred for 10 minutes and then poured into 250 mL 1M HCl, forming an orange precipitate. The mixture was extracted with CHCl _3_ (3x), the organic phase dried with Na _2_SO _4_ and the solvent removed under reduced pressure. The crude product was recrystallised from MeCN, yielding **S-P2** as an orange solid (630 mg, 2.8 mmol, 82%). ^1^H NMR (400 MHz, CDCl _3_): δ 10.41 (s, 2H), 7.80 (s, 2H), 2.57 (s, 6H) ppm; in accordance with the literature ^9^.

**Methoxy-substituted [2.2.2.2]paracyclophanetetraene O-PCT:** Wittig cyclisation precursor **O-P1** (1.19 g, 1.4 mmol, 1.0 equiv.) and terephthalaldehyde ( **P2**) (188 mg, 1.4 mmol, 1.0 equiv.) were dissolved in 70 mL anhydrous DMF under nitrogen, purged with nitrogen for 5 min and cooled to -40 °C using an MeCN/dry ice bath. Lithium methoxide (159 mg, 4.2 mmol, 3.0 equiv.) was dissolved in 18 mL anhydrous MeOH by sonication, purged with nitrogen for 2 minutes (purge carefully to avoid precipitation) and added to the reaction over 9 hours using a syringe pump. The rate of addition was adjusted to maintain a faint red colour of the solution. After complete addition of the base, the reaction was stirred overnight, using a magnetic stirrer, while warming to r.t. The resulting suspension was poured into H _2_O and extracted with Et _2_O (3x), the organic phase dried with Na _2_SO _4_ and the solvent removed in vacuo. The obtained solid was dissolved CH _2_Cl _2_ and flashed over silica using CH _2_Cl _2_ as the eluent until the solvent ran clear (to remove the Wittig reaction side product triphenylphosphine oxide (TPPO)). The crude product was purified by recycling preparative GPC using CHCl _3_ as the eluent, yielding **O-PCT** as a yellow oil that crystallised slowly over time (32 mg, 0.06 mmol, 9%). ^1^H NMR (400 MHz, CDCl _3_): δ 7.08 (s, 8H), 6.72 (s, 4H), 6.58 (d, *J* = 12.2 Hz, 4H), 6.52 (d, *J* = 12.1 Hz, 4H), 3.55 (s, 12H) ppm. ^13^C{ ^1^H} NMR (101 MHz, CDCl _3_): δ 151.1, 136.0, 130.8, 128.8, 127.0, 126.2, 113.4, 56.3 ppm. HRMS ( *m/z*): [M] ^+^ calcd for C _36_H _32_O _4_: 528.2301, found: 528.2301 (ESI).

**Methylthio-substituted [2.2.2.2]paracyclophanetetraene S-PCT:** Wittig cyclisation precursor **S-P1** (1.00 g, 1.14 mmol, 1.0 equiv.) and terephthalaldehyde ( **P2**) (153 mg, 1.14 mmol, 1.0 equiv.) were dissolved in 35 mL anhydrous DMF under nitrogen, purged with nitrogen for 5 min and cooled to -40 °C using an MeCN/dry ice bath. Lithium methoxide (130 mg, 3.4 mmol, 3.0 equiv.) was dissolved in 9 mL anhydrous MeOH by sonication, purged with nitrogen for 2 minutes (purge carefully to avoid precipitation) and added to the reaction over 9 hours using a syringe pump. The rate of addition was adjusted to maintain a faint red colour of the solution. After complete addition of the base, the reaction was stirred overnight while warming to r.t. The resulting suspension was poured into H _2_O and extracted with Et _2_O (3x), the organic phase dried with Na _2_SO _4_ and the solvent removed in vacuo. The obtained solid was dissolved CH _2_Cl _2_ and flashed over silica using CH _2_Cl _2_ as the eluent until the solvent ran clear (to remove the Wittig reaction side product triphenylphosphine oxide (TPPO)). The crude product was purified by recycling preparative GPC using CHCl _3_ as the eluent, yielding **S-PCT** as an orange solid (48 mg, 0.08 mmol, 14%). ^1^H NMR measurements (including measurements at elevated temperature) suggest that the product is a mixture of two rotamers (ratio approx. 10:1, determined from ^1^H NMR integrals). *Main rotamer:* ^1^H NMR (400 MHz, CDCl _3_): δ 6.97 (s, 4H), 6.95 (s, 8H), 6.63 (d, *J* = 12.0 Hz, 4H), 6.57 (d, *J* = 12.0 Hz, 4H), 2.14 (s, 12H) ppm. ^13^C{ ^1^H} NMR (101 MHz, CDCl _3_): δ 137.0, 135.6, 134.2, 131.5, 129.0, 128.2, 127.0, 16.0 ppm. ^1^H NMR (400 MHz, CDCl _3_, 50 °C): δ 7.00 (s, 4H), 6.95 (s, 8H), 6.65 (d, *J* = 11.9 Hz, 4H), 6.58 (d, *J* = 11.9 Hz, 4H), 2.14 (s, 12H) ppm. *Minor rotamer:* ^1^H NMR (400 MHz, CDCl _3_): 7.00 (s, 4H), 6.98 (s, 8H), 6.58 (s, 8H), 2.22 (s, 12H) ppm. ^1^H NMR (400 MHz, CDCl _3_, 50 °C): δ 7.03 (s, 4H), 6.99 (s, 8H), 6.58 (s, 8H), 2.21 (s, 12H) ppm. HRMS ( *m/z*): [M+H] ^+^ calcd for C _36_H _32_S _4_: 593.1460, found: 593.1444 (APCI).

### Measurement methods and instrumentation

NMR spectra were recorded in CDCl _3_ solution at 400 MHz for ^1^H, 101 MHz for ^13^C, and 162 MHz for ^31^P on a Bruker AV-400 spectrometer. High-resolution mass spectrometry (HRMS) was carried out on systems from Thermo Scientific (Thermo Scientific Q-Exactive/Dionex Ultimate 3000) for atmospheric pressure chemical ionization (APCI) and Waters (Waters LCT Premier (ES-ToF)/Acquity i-Class) for electrospray ionization (ESI). While the Thermo Scientific system gives the actual mass of the ionized compounds, the Waters system is calibrated to give the mass of the neutral compounds. This was considered when calculating the *m/z* values for comparison with the measurements.

UV-vis absorption spectra were recorded on an Agilent Cary 60 UV-vis spectrophotometer at room temperature. The measurements were carried out with 5 μM solutions in CHCl _3_ at a scan rate of 300 nm min ^-1^ and a data interval of 0.5 nm. The baseline was corrected for plotting the data in Figure 5. Photoluminescence (PL) spectra were acquired on an Agilent Cary Eclipse fluorescence spectrophotometer with 5 μM solutions in CHCl _3_ at a scan rate of 120 nm min ^-1^ and a data interval of 1 nm. The excitation and emission slits were set to 5 nm, the emission and excitation filters were set to ‘auto’ setting, and the detector voltage was set to ‘high’ (800 V). To facilitate a comparison, these are the same settings as used in our previous work on a set of ester-substituted macrocycles ^6^.

Cyclic voltammetry (CV) measurements were carried out at arbitrary concentration using a glassy carbon working electrode, a platinum mesh auxiliary electrode, and a silver wire quasi-reference electrode (QRE) at a scan rate of 0.1 V s ^-1^. 0.1 M Tetrabutylammonium hexafluorophosphate (NBu _4_PF _6_) in dichloroethane (DCE) or dimethylformamide (DMF) were used as the supporting electrolyte solution. Ferrocene (Fc) was measured as reference. The solutions were purged with nitrogen for 5 min prior to the measurements. However, the presences of a reduction process at around -1.4 V vs. Fc/Fc ^+^ in the measurements suggests that some residual oxygen was not efficiently removed by the purging process ^20^. This reduction process was also present when measuring the supporting electrolyte solutions only (without a sample), corroborating that the process does not involve the macrocycles. In line with best practice, the redox potentials were estimated from the half-wave potential (E ^1/2^) when reversibility was observed and from the inflection-point potential (E ^i^) when no reversibility was observed ^21^.

### Computational methods

Geometries for the neutral S _0_ and T _1_ states as well as the dianion and dication were optimized in vacuum using density functional theory (DFT) with the PBE0 functional ^22, 23^ along with the def2-SV(P) basis set ^24^ and the D3 dispersion correction ^25^ in its optimised power version ^26^. Transition state (TS) energies were optimized via a constrained optimization, fixing two of the phenylene-vinylene torsion angles, considering that a full TS optimization did not converge for **S-PCT** and verifying that the energies between this method and the full optimization were consistent for **O-PCT**. Interconversion times (t) between rotamers were estimated via the Eyring equation


$$\frac{1}{t}\;=\;\frac{kT}{h}\;\exp\;(-\frac{\Delta_{a}E}{kT})$$

where *k* and *h* are the Boltzmann and Planck constants, T = 298 K is the temperature and Δ _a_ *E* is the computed activation barrier. Redox potentials were computed using the PBE0 functional along with the def2-SVPD basis set using a conductor-like polarizable continuum model ^27^ considering a dielectric constant of 10.125 to represent 1,2-dichloroethane (DCE) and following the procedure described in detail in Ref. 6. All these computations were carried out in Q-Chem 5.3 ^28, 29^. S _1_ states were optimised using time-dependent (TD) DFT with the ωPBEh functional ^30^ (using ω=0.1 a.u. and 20% global Hartree-Fock exchange) along with the def2-SV(P) basis set and the D3 dispersion correction using the same D3-parameters as for PBE0.

Chemical shielding tensors were computed at the PBE0/def2-SVP level using gauge including atomic orbitals ^31^ as implemented in Gaussian 09 ^32^. Shielding tensors were represented graphically using the VIST (visualisation of chemical shielding tensors) method ^14^ as implemented in TheoDORE 2.4 ^33^ using cclib ^34^ for some of the file parsing work and VMD ^35^ for the final graphical representation.

NDOs ^16^ for the T _1_ and charged states were computed using TheoDORE following two independent DFT calculations. NDOs for the S _1_ state were computed directly within Q-Chem after the TDDFT computation.

## Data availability

### Underlying data

Zenodo: Research data for "[2.2.2.2]Paracyclophanetetraenes (PCTs): cyclic structural analogues of poly(p-phenylene vinylene)s (PPVs)". https://doi.org/10.5281/zenodo.5206265 ^36^

This project contains the following data:

- The underlying experimental research data ( ^1^H NMR, ^13^C NMR, ^31^P NMR, high-resolution mass spectrometry (HRMS), UV-vis absorption, photoluminescence (PL), cyclic voltammetry)
- The underlying computational research data (molecular geometries, input/output files for Q-Chem, Gaussian, and TheoDORE)

Data are available under the terms of the Creative Commons Attribution 4.0 International license (CC-BY 4.0).

The ^1^H NMR, ^13^C NMR, and ^31^P NMR spectra are also available via ChemSpider. See CSIDs: 120300 ( **S-2**), 3332510 ( **O-3**), 110417237 ( **S-3**), 110417244 ( **O-P1**), 110417245 ( **S-P1**), 14757439 ( **Br-P2**), 110417240 ( **S-P2**), 110417241 ( **O-PCT**), and 110417242 ( **S-PCT**).
