# Supplementary material for: [2.2.2.2]Paracyclophanetetraenes (PCTs): cyclic structural analogues of poly( p‑phenylene vinylene)s (PPVs)
Source: Open Res Eur. 2022 Mar 10;1:111. Originally published 2021 Sep 23. [Version 2] doi: 10.12688/openreseurope.13723.2 (PMC10445936; doi:10.12688/openreseurope.13723.2)
Supplement: Peer review history

# Peer review: Reviewer response for version 2

**DOI:** 10.21956/openreseurope.15764.r28785

**Published:** 2022-04-05

**Authors:** Miłosz Pawlicki

**Affiliations:** 1 Department of Chemistry, Jagiellonian University, Kraków, Poland

The revised manuscript of Glöcklhofer and co-workers addresses all crucial points gathered in both previous reports and currently can be accepted without reservations. I highly appreciate the presented discussion on the fluorescence quantum yields and extended presentation of the magnetic properties. As mentioned in my previous report it is important to compare the descriptors of diatropic/paratropic behaviour what directs us to more comprehensive picture of aromaticity/antiaromaticity and the properties of those systems.

Is the study design appropriate and does the work have academic merit?

Yes

Is the work clearly and accurately presented and does it cite the current literature?

Yes

If applicable, is the statistical analysis and its interpretation appropriate?

Not applicable

Are all the source data underlying the results available to ensure full reproducibility?

Yes

Are the conclusions drawn adequately supported by the results?

Partly

Are sufficient details of methods and analysis provided to allow replication by others?

Yes

Reviewer Expertise:

Organic synthesis, pi-conjugation, aromaticty, spectroscopy,

I confirm that I have read this submission and believe that I have an appropriate level of expertise to confirm that it is of an acceptable scientific standard.

# Peer review: Reviewer response for version 1

**DOI:** 10.21956/openreseurope.14798.r28154

**Published:** 2022-02-14

**Authors:** Miłosz Pawlicki

**Affiliations:** 1 Department of Chemistry, Jagiellonian University, Kraków, Poland

The presented material shows a specific construction of cyclic motifs incorporating p-phenylenes substituted with heteroatoms - oxygen or sulfur. All the conclusions are focusing on the observed changes in conjugation triggered by the excitation but are also theoretically analysed for redox modified structures. 

 The dynamic process recorded within the skeleton (Fig .4) can have a significant influence on the emission efficiency. I would recommend an extension of the scope of experiments with a viscosity dependent measurements and checking is there any influence of the dynamic behaviour on the final fluorescence outcome. The dynamic of the excited state (S1) is very sensitive to several factors including a change of aromatic character mentioned by the Authors but it also indicates the stability of the excited state in addition to adding another proof for the observed behaviour. I would suggest extending the discussion by this data that will be beneficial for a deeper understanding of observed properties. The second crucial parameter for fluorescence observed within any molecule is a quantum yield which has not been included in the presented material. Extension of the analysis on those two key parameters for all reported systems will create a comprehensive picture of the conjugation observed in those structural motifs.

 The presented material discusses two different isomers that differed by the rotation of disubstituted phenylene but can the described dynamic process be treated as a simple rotation? While looking at the proximity of present substituents it potentially can be analysed as a concerted process where a move of one substituted phenylenes will cause a deep change in the whole system forcing also the move of unsubstituted phenylenes. I would recommend an extension of this discussion by incorporation of additional data explaining the isomerisation which will bring the Reader a set of conformational changes necessary for observing switching from one isomer to another.

 The theoretical analysis of changes in delocalisation relies on the VIST analysis - an original approach for visuliation of the changes in aromatic/antiaromatic character developed by the Authors of this manuscript. I would suggest to compare the observed behaviour with any other theoretical descriptors of aromaticity/antiaromaticity (e.g. NICS(0,1,2)) which will eventually bring a comprehensive picture. 

 In conclusion, the presented material brings insight to macrocyclic systems constructed with p-phenylenes showing modulation of conjugation within those systems potentially beneficial for several fields.

Is the study design appropriate and does the work have academic merit?

Yes

Is the work clearly and accurately presented and does it cite the current literature?

Yes

If applicable, is the statistical analysis and its interpretation appropriate?

Not applicable

Are all the source data underlying the results available to ensure full reproducibility?

Yes

Are the conclusions drawn adequately supported by the results?

Partly

Are sufficient details of methods and analysis provided to allow replication by others?

Yes

Reviewer Expertise:

Organic synthesis, pi-conjugation, aromaticty, spectroscopy,

I confirm that I have read this submission and believe that I have an appropriate level of expertise to confirm that it is of an acceptable scientific standard, however I have significant reservations, as outlined above.

# Response

**Published:** 2022-03-03

**Authors:** Florian Glöcklhofer

**Affiliations:** 21686, UK

Thank you for reviewing and approving our article with reservations. We would like to respond to the comments below and hope that with the changes made in response the article can be approved for indexing in bibliographic databases. (Reviewer comments in italics)

*The dynamic process recorded within the skeleton (Fig .4) can have a significant influence on the emission efficiency. I would recommend an extension of the scope of experiments with a viscosity dependent measurements and checking is there any influence of the dynamic behaviour on the final fluorescence outcome. The dynamic of the excited state (S1) is very sensitive to several factors including a change of aromatic character mentioned by the Authors but it also indicates the stability of the excited state in addition to adding another proof for the observed behaviour. I would suggest extending the discussion by this data that will be beneficial for a deeper understanding of observed properties. The second crucial parameter for fluorescence observed within any molecule is a quantum yield which has not been included in the presented material. Extension of the analysis on those two key parameters for all reported systems will create a comprehensive picture of the conjugation observed in those structural motifs.*

**Response: **We thank the reviewer for these recommendations. We have now determined the PLQYs of the macrocycles using quinine in 0.05 M H _2_SO _4_ as the reference to further support the conclusions of the article. Despite the observed increase in PL intensity upon introduction of the substituents, all three investigated macrocycles are weak emitters; PLQYs of 0.06% for **PCT**, 0.65% for **O-PCT**, and 0.56% for **S-PCT** were determined. Due to the large difference in PLQY between the reference and the macrocycles, neutral density filters had to be used to enable PL measurements of the reference under the same instrument settings as used for the macrocycles. We believe this relative determination of the PLQYs can provide a good indication of the order of magnitude, but we suggest refraining from an overinterpretation of the exact values due to the potential sources of error in the determination. We have added the following sentence in the results and discussion section of the article and updated the conclusions and methods sections as well as the provided underlying research data accordingly: “Despite the increase in PL intensity upon introduction of the substituents, all three macrocycles are still weak emitters, with a relative determination of the photoluminescence quantum yield (PLQY) indicating values of  <1% for **O-PCT** and **S-PCT** and <0.1% for **PCT**.” We agree that viscosity dependent measurements could provide even further insights and conclusions. Nevertheless, we believe that the current conclusions of the article are adequately supported by the provided results and that further photophysical measurements are beyond the scope of this article. The article does not intend to provide a detailed photophysical study of the synthesised macrocycles but, in addition to the synthesis and computational analysis of the macrocycles, aims to provide some basic photophysical and electrochemical characterisation as well as a comparison of the characterisation results obtained for the different macrocycles. From the careful language used in the discussion of the photoluminescence intensities (“t entatively assigned to symmetry breaking in the excited state”, “the spectra suggest that symmetry breaking is only a dynamic effect”) and in the conclusions (“a feature that we assign to dynamic excited-state symmetry breaking”), we believe it should be clear that further photophysical studies are required for a better understanding of the effects and a more definitive explanation. We have added “tentatively” in the conclusions to further clarify this there.

*The presented material discusses two different isomers that differed by the rotation of disubstituted phenylene but can the described dynamic process be treated as a simple rotation? While looking at the proximity of present substituents it potentially can be analysed as a concerted process where a move of one substituted phenylenes will cause a deep change in the whole system forcing also the move of unsubstituted phenylenes. I would recommend an extension of this discussion by incorporation of additional data explaining the isomerisation which will bring the Reader a set of conformational changes necessary for observing switching from one isomer to another.*

**Response:** We agree with the reviewer that the switch between the two isomers is a non-trivial dynamical process. Indeed, it was challenging to find a well-defined reaction coordinate for this process. The purpose of the computations performed was to illustrate the qualitative difference between the reaction barriers to explain the NMR signals. A full mechanistic study of the interconversion process is out of the scope of this study.

*The theoretical analysis of changes in delocalisation relies on the VIST analysis - an original approach for visuliation of the changes in aromatic/antiaromatic character developed by the Authors of this manuscript. I would suggest to compare the observed behaviour with any other theoretical descriptors of aromaticity/antiaromaticity (e.g. NICS(0,1,2)) which will eventually bring a comprehensive picture.*

**Response: **Following the reviewer’s suggestion, we have added NICS values in the paper, see Table 1. Indeed the NICS value printed is directly related to the size of the dumbbell in the VIST plot.

# Peer review: Reviewer response for version 1

**DOI:** 10.21956/openreseurope.14798.r28148

**Published:** 2021-12-23

**Authors:** Weixuan Zeng

**Affiliations:** 1 Department of Chemistry, University of Cambridge, Cambridge, UK

The manuscript by Pletzer and co-workers described a combined experimental and computational study on the methoxy- and methylthio-substituented [2.2.2.2]paracyclophanetetraene (PCT). The rotamers cased by the substituents not only changed the geometries but also the symmetry properties of the compounds, which resulted in significant changes in their excited-state aromaticity features. The reviewer thought this work well extended the previous study on the ‘PCT’ series by the authors and got some interesting results for readers, and therefore reaches the acceptable standard as this form.

 Here’re some recommendations for further improvement:

1. More accurate concentration of the solution samples for the UV-Vis and PL measurements is recommended to be provided. Further discussion on their photoluminescent quantum yields instead of the comparison of the intensity would be more suitable for the discussion about the photophysics characters of the compounds.
2. The chemical shift features as aromaticity criteria might be very sensitive to the geometry changes. Significant geometries change can be found in the S-PCT Cs series, which can’t clearly differentiate the contribution on its aromaticity mainly from the symmetry breaking. Some more theoretical models are recommended here, the authors could introduce hydroxy- and sulfydryl-substituents to reduce the steric hindrance. And substituents on the parent PCT models without further relaxation are recommended for comparison.

Is the study design appropriate and does the work have academic merit?

Yes

Is the work clearly and accurately presented and does it cite the current literature?

Yes

If applicable, is the statistical analysis and its interpretation appropriate?

Yes

Are all the source data underlying the results available to ensure full reproducibility?

Yes

Are the conclusions drawn adequately supported by the results?

Yes

Are sufficient details of methods and analysis provided to allow replication by others?

Yes

Reviewer Expertise:

NA

I confirm that I have read this submission and believe that I have an appropriate level of expertise to confirm that it is of an acceptable scientific standard.

# Response

**Published:** 2022-03-03

**Authors:** Florian Glöcklhofer

**Affiliations:** 21686, UK

Thank you for reviewing and approving our article and providing recommendations for further improvement. We would like to respond to these recommendations below. (Reviewer comments in italics)

- *More accurate concentration of the solution samples for the UV-Vis and PL measurements is recommended to be provided. Further discussion on their photoluminescent quantum yields instead of the comparison of the intensity would be more suitable for the discussion about the photophysics characters of the compounds.*

**Response: **The UV-vis and PL measurements were performed using 5 μM solutions in CHCl _3_, as stated in the methods section. If the reviewer’s comment is referring to “approx. 5 μM” in the caption of Figure 5, we would like to point out that “approx.” was meant to reflect the limitations in accuracy when working with milligram quantities (despite using a high-precision balance). We have removed “approx.” from the caption to avoid the impression that the concentration was not as accurate as possible. We appreciate the suggestion regarding the photoluminescence quantum yields (PLQYs). We have now determined the PLQYs of the macrocycles using quinine in 0.05 M H _2_SO _4_ as the reference to further support the conclusions of the article. Despite the observed increase in PL intensity upon introduction of the substituents, all three investigated macrocycles are still weak emitters; PLQYs of 0.06% for **PCT**, 0.65% for **O-PCT**, and 0.56% for **S-PCT** were determined. Due to the large difference in PLQY between the reference and the macrocycles, neutral density filters had to be used to enable PL measurements of the reference under the same instrument settings as used for the macrocycles. We believe this relative determination of the PLQYs can provide a good indication of the order of magnitude, but we suggest refraining from an overinterpretation of the exact values due to the potential sources of error in the determination. We have added the following sentence in the results and discussion section of the article and updated the conclusions and methods sections as well as the provided underlying research data accordingly: “Despite the increase in PL intensity upon introduction of the substituents, all three macrocycles are still weak emitters, with a relative determination of the photoluminescence quantum yield (PLQY) indicating values of  <1% for **O-PCT** and **S-PCT** and <0.1% for **PCT**.”

- *The chemical shift features as aromaticity criteria might be very sensitive to the geometry changes. Significant geometries change can be found in the S-PCT Cs series, which can’t clearly differentiate the contribution on its aromaticity mainly from the symmetry breaking. Some more theoretical models are recommended here, the authors could introduce hydroxy- and sulfydryl-substituents to reduce the steric hindrance. And substituents on the parent PCT models without further relaxation are recommended for comparison.*

**Response: **We thank the reviewer for this suggestion. We have included computations on **O-PCT** and **S-PCT** based on a frozen **PCT** structure, see Table 1 and the ensuing discussion. Indeed, this procedure leads to similar NICS values as seen in the unsubstituted **PCT** molecule.
